# Machine performance and stability of the first clinical self‐shielded stereotactic radiosurgery system: Initial 2‐year experience

**DOI:** 10.1002/acm2.13857

**Published:** 2022-12-15

**Authors:** Shiv P. Srivastava, Stephen P. Sorensen, Shyam S. Jani, Xiangsheng Yan, Dilini S. Pinnaduwage

**Affiliations:** ^1^ Department of Radiation Oncology St. Joseph's Hospital and Medical Center Phoenix AZ USA

**Keywords:** beam output, DQA, gafchromic film, ion chamber, machine performance, targeting, ZAP‐X

## Abstract

This study provides insight into the overall system performance, stability, and delivery accuracy of the first clinical self‐shielded stereotactic radiosurgery (SRS) system. Quality assurance procedures specifically developed for this unit are discussed, and trends and variations over the course of 2‐years for beam constancy, targeting and dose delivery are presented. Absolute dose calibration for this 2.7 MV unit is performed to deliver 1 cGy/MU at d_max _= 7 mm at a source‐to‐axis‐distance (SAD) of 450 mm for a 25 mm collimator. Output measurements were made with 2‐setups: a device that attaches to a fixed position on the couch (daily) and a spherical phantom that attaches to the collimating wheel (monthly). Beam energy was measured using a cylindrical acrylic phantom at depths of 100 (D_10_) and 200 (D_20_) mm. Beam profiles were evaluated using Gafchromic film and compared with TPS beam data. Accuracy in beam targeting was quantified with the Winston‐Lutz (WL) and end‐to‐end (E2E) tests. Delivery quality assurance (DQA) was performed prior to clinical treatments using Gafchromic EBT3/XD film. Net cumulative output adjustments of 15% (pre‐clinical), 9% (1st year) and 3% (2nd year) were made. The mean output was 0.997 ± 0.010 cGy/MU (range: 0.960–1.046 cGy/MU) and 0.993 ± 0.029 cGy/MU (range: 0.884–1.065 cGy/MU) for measurements with the daily and monthly setups, respectively. The mean relative beam energy (D_10_/D_20_) was 0.998 ± 0.004 (range: 0.991–1.006). The mean total targeting error was 0.46 ± 0.17 mm (range: 0.06–0.98 mm) for the WL and 0.52 ± 0.28 mm (range: 0.11–1.27 mm) for the E2E tests. The average gamma pass rates for DQA measurements were 99.0% and 90.5% for 2%/2 mm and 2%/1 mm gamma criteria, respectively. This SRS unit meets tolerance limits recommended by TG‐135, MPPG 9a., and TG‐142 with a treatment delivery accuracy similar to what is achieved by other SRS systems.

## 1 INTRODUCTION

The ZAP‐X (Zap Surgical Systems, Inc., San Carlos, CA, USA) is a novel stereotactic radiosurgery (SRS) delivery system intended for the treatment of intracranial and upper cervical spine lesions.^1^ The first clinical ZAP‐X unit was installed at Barrow Brain and Spine at St. Joseph's Hospital and Medical Centre (Phoenix, AZ, USA), and has been in clinical use since January 2019. This is the first study to report on the stability and performance of this unit over the first 2 years of clinical use.

**FIGURE 1:**
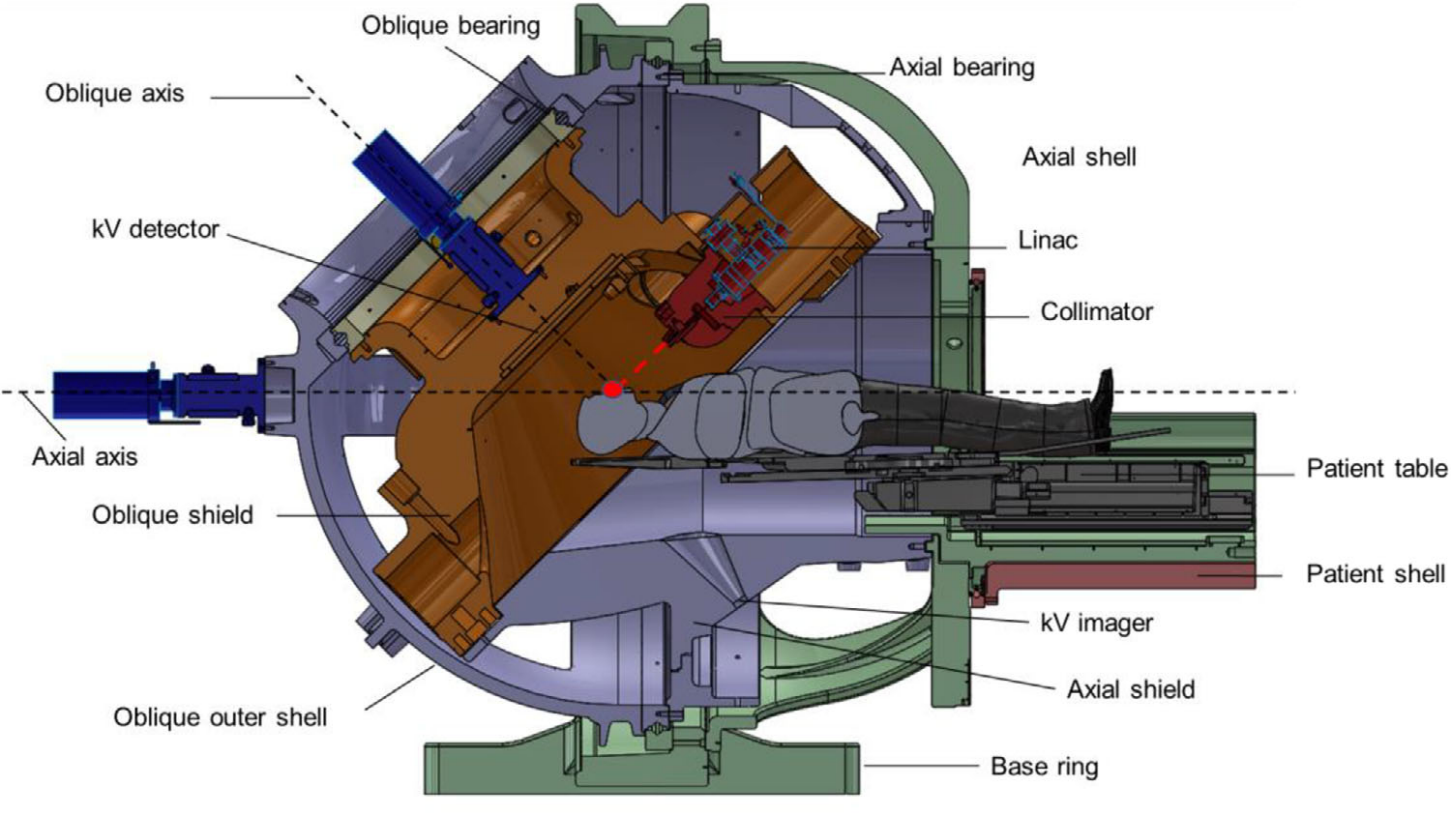
A schematic diagram of the ZAP‐X stereotactic radiosurgery system. Fixed components are shown in green. Axial rotating components are shown in purple and components that can rotate both in the axial and oblique planes are shown in brown. (*Image courtesy of Zap Surgical*)

The self‐shielded ZAP‐X system design comprises of a shielded treatment chamber, rotating shell (Figure 1) and a pneumatic door located at the foot of the table. This unique shielding design reduces radiation exposure levels inside the treatment room to well within those recommended by the National Council on Radiation Protection and Measurements (NCRP) for radiation workers,^2^, ^3^, ^4^ thereby eliminating the need for a shielded vault while increasing installation flexibility. This allows the staff (therapists, radiation oncologists, neurosurgeons, physicists, nurses etc.) to administer treatment while remaining within the treatment vault. Radiation exposure limits for our unit were independently verified by Sorensen et al.^5^ and appropriate workload recommendations were established prior to the unit's clinical use.

The radiation source for the ZAP‐X is a 2.7 MV S‐band linear accelerator mounted on a pair of connected gimbals that allows for isocentric, non‐coplanar, dual‐axis radiation delivery over a solid angle of over 2π steradians, comparable to the delivery space offered by other dedicated SRS systems. The flattening filter free beam is collimated via a rotating tungsten wheel with eight circular field sizes ranging from 4 mm to 25 mm in diameter defined at a source‐to‐axis distance (SAD) of 450 mm. In initial work,^6^ the ZAP‐X produced the lowest full‐width half maximum and sharpest overall penumbra for its smallest collimator compared to beam profile parameters of a Gamma Knife (GK) and CyberKnife (CK) for the corresponding smallest collimator. Pre‐treatment positioning and intrafraction monitoring for the ZAP‐X is performed through a combination of robotic couch movements (pitch, yaw, and superior/inferior) and an internally‐mounted kilovoltage (kV) imaging system that compares real‐time X‐ray images to digitally reconstructed radiographs (DRRs) from the treatment planning system (TPS). A megavoltage detector is available for measurement of exit dosimetry for each beam, allowing for real‐time treatment delivery verification.^7^

The stability and performance of an SRS unit is indicative of its ability to deliver accurate treatments. Critical components of overall system performance include beam output, energy, profile constancy, targeting, dose delivery accuracy, and on‐board imager accuracy/quality. The American Association of Physicists in Medicine (AAPM) has Task Groups (TG) specifically addressing periodic quality assurance (QA) procedures and tolerance limits for SRS units to assess and ensure the stability and performance of these systems.^8^, ^9^ Our institution has an active radiosurgery practice with multiple SRS delivery units available for the treatment of brain lesions, including GK, CK and conventional linear accelerators, in addition to the ZAP‐X. Within the first 2‐years, the ZAP‐X was used for 89 treatments including patients with brain metastasis, meningiomas, pituitary adenocarcinomas, hemangioblastomas and glioblastomas. Here, we provide a comprehensive report on the periodical QA procedures performed for the ZAP‐X by our group. Further, this report for the first time provides insight into the stability and performance of the ZAP‐X by presenting trends in machine performance for important dosimetric, mechanical and imaging components, providing a useful reference for current and future ZAP‐X users.

## 2 METHODS

Following installation of the ZAP‐X, acceptance testing was performed per vendor recommended guidelines. Comprehensive commissioning^10^, ^11^, ^12^ and validation^13^ work was done using the published guidelines from TG‐106,^14^ TG‐53,^15^ Medical Physics Practice Guidelines (MPPG) 5.a,^16^ International Atomic Energy Agency's (IAEA) Technical Report Series (TRS)‐430^17^ and TRS‐398.^18^

### 2.1 Beam stability

#### 2.1.1 Beam output measurements

Absolute dose calibration was performed according to the TG‐21^19^ protocol using a spherical acrylic phantom developed by Sorensen et al.,^12^ and a PTW TN31010 (0.125 cc Semiflex) ionization chamber (IC). Details regarding the use of a modified TG‐21 approach for dose calibration similar to what is done for Gamma Knife,^20^, ^21^ and its validation, have been addressed in a previous publication.^12^ The beam output was calibrated to deliver 1.0 cGy per monitor unit (MU) at the depth of maximum dose (d_max _= 7 mm) for a SAD of 450 mm using the 25 mm collimator as the reference field size. The nominal dose rate was 1500 cGy/min. This absolute dose calibration was verified using a modified TG‐51^22^ protocol and independently verified via an Imaging and Radiation Oncology Core (IROC) phantom irradiation.

##### Daily output measurements

**FIGURE 2:**
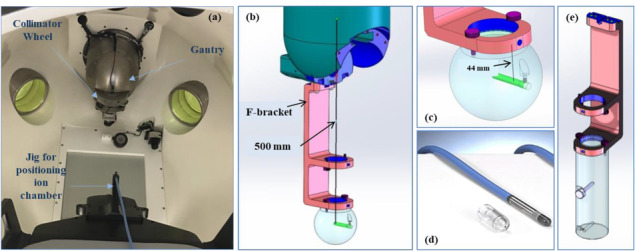
(a). Daily setup for output measurements where the ion camber is secured in a jig that attaches to the couch. (b). A schematic of the monthly setup for output measurements where the spherical phantom is attached to the ‘F‐bracket’ which in turn attaches the collimating wheel. (c). A close‐up of the spherical phantom showing the opening for the ion chamber in green. (d). The 0.125 cc PTW SemiFlex ion chamber used for output measurements. (e). A schematic of the cylindrical phantom attached to the ‘F‐bracket’ used for beam energy measurements. *(Images 2b, c and d are courtesy of Zap Surgical. Image 2d is used with permission from PTW)*.

Daily output measurements were performed using a QA device (Figure 2a) provided by the vendor. The base of this device attaches to a fixed position on the head of the couch and allows for a PTW 31010 SemiFlex ion chamber to be inserted in the opening at the top of the QA device. The couch is then sent to a preset position which places the ion chamber with the buildup cap on, at isocentre. Output measurements are made with the gantry at ‘North Pole’ (0^0^ position) for the 25 mm collimator after proper warm up (5000 MU delivered prior to measurements) delivering 100 MU for 3 charge measurements acquired with the electrometer bias set at ‐300 V. A correction for temperature and pressure was applied to the average charge reading and a charge‐to‐dose (cGy/nC) conversion factor was applied based on daily output measurements done immediately following the absolute dose calibration. The pressure was measured with a handheld barometer and the temperature was measured by placing a lollipop thermometer on the couch in the near vicinity of the IC. Daily output measurements were performed by a trained therapist.

##### Monthly output measurements

The TG‐21 based approach mentioned earlier^19^ was used for monthly output measurements. In this setup, a near spherical acrylic phantom was attached to the bottom of the vendor provided F‐bracket (Figure 2b). This acrylic phantom design was specifically developed by Sorensen et al.,^12^ to accommodate a PTW 31010 SemiFlex IC (Figure 2c) at a depth of 44 mm (equivalent to 50 mm depth in water) at 500 mm SAD (Figures 2b and c) to meet TG‐21^19^ dose calibration specifications for a 3 MV beam when using an IC with an inner diameter of 5.5 mm. The F‐bracket itself attaches to the collimator head. Output measurements were made by a qualified medical physicist by delivering three separate measurements of 100 MU following machine warm up (5000 MU) with a collimator opening of 25 mm and the gantry positioned at ‘North Pole’. The ambient pressure was measured with a handheld barometer and the temperature was measured by placing a lollipop thermometer in the spherical phantom's IC insert while mounted on the F‐bracket. Barometer and thermometer were calibrated annually. Charge measurements were taken using PTW T10010 electrometer setting at −300 V and the average reading was corrected for temperature and pressure, ion recombination and polarity effects. An inverse square correction was used to obtain the dose per monitor unit at d_max_ (7 mm) at 450 mm SAD, using the clinical depth dose measurements acquired in water at 50 mm (450 SSD).

##### Annual output measurements

Annual output measurements were made using the same setup as that described above for monthly measurements. However, these measurements were independently verified using a modified TG‐51^22^ approach. Briefly, TG‐51 measurements were carried out using the MP3‐XS water phantom (PTW‐Freiberg, Freiberg, Germany) with the geometric centre of a ADCL calibrated SemiFlex IC (with an absorbed dose to water calibration factor, $N_{D,w}^{60_{Co}}$ = 3.006 × 10^8^ Gy/C) placed at 50 mm depth in water with the SSD = 450 mm for the 25 mm collimator.^12^ The beam quality conversion factor ($k_{Q}$) for the SemiFlex IC was taken to be 1.000 given that the average energy of the ZAP‐X is comparable to that of Co‐60 and based on preliminary work carried out by Townson et al.^23^ Corrections for ion recombination, polarity, electrometer and temperature and pressure were applied to the average of three measured readings acquired by delivering 100 MU each, following proper machine warm up. The percentage depth dose curve for TG‐51 was acquired using a PTW 60012 diode accounting for the diode effective point of measurement with a 450 mm SSD. Details regarding the process for water tank setup in the ZAP‐X unit and percentage depth dose data acquisition, has been previously reported by Pinnaduwage et al.^10^ An inverse square distance correction was applied to the corrected charge measurement to obtain dose per monitor unit at d_max_ at 450 mm SAD. Peak scatter factors (PSFs) provided by the vendor using previously published reports^24^, ^25^ range from 0.988 to 1.002 (for field sizes ranging from 0‐ to 30‐ mm). Therefore, the ratio of PSFs accounting for effective field size (25.00 mm vs. 25.38 mm) is near unity.

Annual beam output measurements were verified using Gafchromic film (EBT3 or XD) measurements as described elsewhere,^12^ and independently verified through IROC using two different phantoms. Prior to clinical use of the ZAP‐X, the IROC SRS head phantom (an anthropomorphic head phantom containing a 1.9 cm diameter spherical target) was scanned using computed tomography (CT) (1 mm slice thickness), and an isocentric plan using the 20 mm collimator with 36 beams was generated in the ZAP TPS to deliver 20 Gy to the spherical target. This phantom which contains two TLD capsules near the target centre and two orthogonal Gafchromic films, was positioned in the ZAP‐X using kV imaging prior to irradiation. The phantom contains a separate imaging insert to be used for CT scanning and phantom positioning prior to irradiation, and a separate dosimetry insert to be used for treatment delivery.

Additionally, during the 2^nd^ year of clinical treatments beam output was independently verified by IROC using a GK acrylonitrile butadiene styrene (ABS) plastic phantom (Elekta Solutions AB, Stockholm, Sweden). The phantom allows the insertion of a cassette with two thermoluminescent dosimeters (TLDs). An isocentric plan with 64 non‐coplanar beams was generated in the ZAP TPS using the 25 mm collimator, on a thin slice (1 mm) CT scan of this phantom. The plan delivered 600 cGy to the TLDs. The phantom was positioned in the ZAP‐X unit using kV imaging to match the CT simulation position, prior to plan delivery.

#### 2.1.2 Beam energy measurements

A vendor provided acrylic cylindrical phantom (Figure 2e) and F‐bracket were used for monthly beam energy measurements. This cylindrical phantom has two machined holes for a SemiFlex IC to be placed at two depths (d = 100 mm and d = 200 mm) along the beam central axis at 450 mm SSD. A plug made of the same material as the phantom is inserted into the unused hole located at d = 100 mm when the d = 200 mm measurements are taken. For the energy measurement, three charge readings were acquired at each depth with the electrometer at ‐300 V bias while delivering 100 MU for each measurement using the 25 mm collimator. The ratio of the average charge reading at d = 100 mm over that at d = 200 mm was calculated (D_10_/D_20_).

#### 2.1.3 Beam profile measurements

**FIGURE 3:**
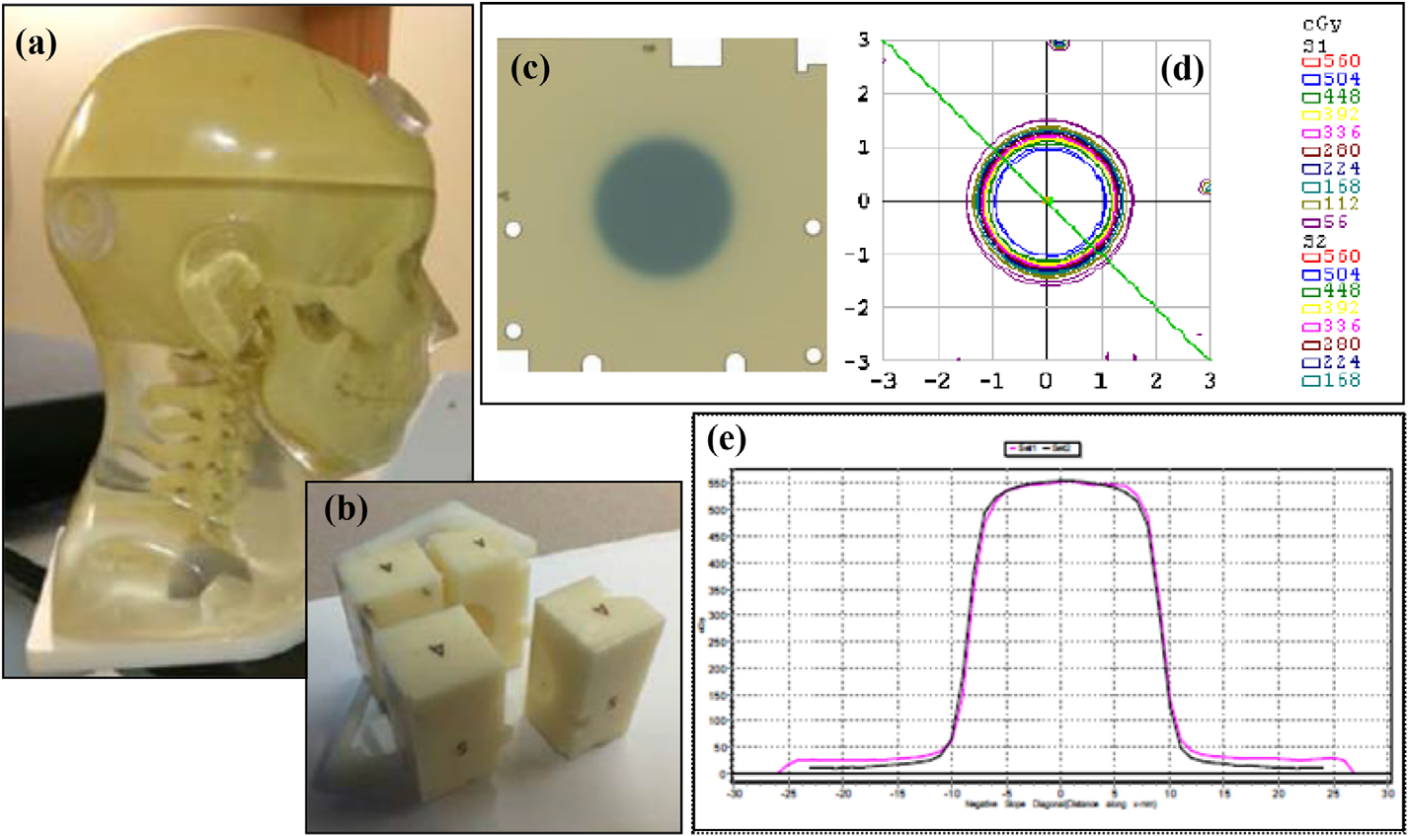
(a). The CyberKnife head phantom and (b) the Ball Cube II insert in which a sagittal no‐slit gafchrmoic film was placed for beam profile measurements. Examples of (c) an irradiated film following delivery of the single beam plan, (d) a dose profile extracted from the treatment planning system and (e) a dose profile comparison used for evaluating the beam shape and symmetry

A sagittal, no slit, Gafchromic EBT3 film (Ashland, Bridgewater, NJ, USA) was placed within the Ball Cube II insert (Figure 3b) in a CK head phantom (Accuray Inc., Sunnyvale, CA, USA) (Figure 3a). The phantom was CT scanned (1 mm slices) with immobilization (head rest and thermoplastic head mask). A single isocentre treatment plan was generated in the ZAP TPS to deliver 600 cGy with the 25 mm collimator, targeting the centre of the hidden target within the Ball Cube with a single lateral beam (gantry at 90^0^). The head phantom was aligned in the ZAP‐X using kV imaging ensuring that the fiducials within the Ball Cube II insert were well aligned between the DRRs and live kV images. The irradiated films (Figure 3c) were scanned using an Epson Expression 10000XL (Seiko Epson Corporation, Nagano, Japan) flatbed scanner (48 bit colour, 75 dpi, no colour corrections applied) following scanner warm up procedures. Film measurements were compared in relative mode with the corresponding sagittal dose plane which was exported from the TPS using SNC patient software (Sun Nuclear, Melbourne, FL, USA). Diagonal beam profiles along the ‘wheelplane’ (measurements in the direction of rotation of the collimator wheel) and ‘orthoplane’ (measurements in the plane perpendicular to the ‘wheelplane’) were compared (Figures 3d and e) to determine constancy in field size, flatness and symmetry. Profile measurements were conducted monthly.

### 2.2 Beam and dose targeting accuracy

#### 2.2.1 Daily Winston‐Lutz (WL) test

**FIGURE 4:**
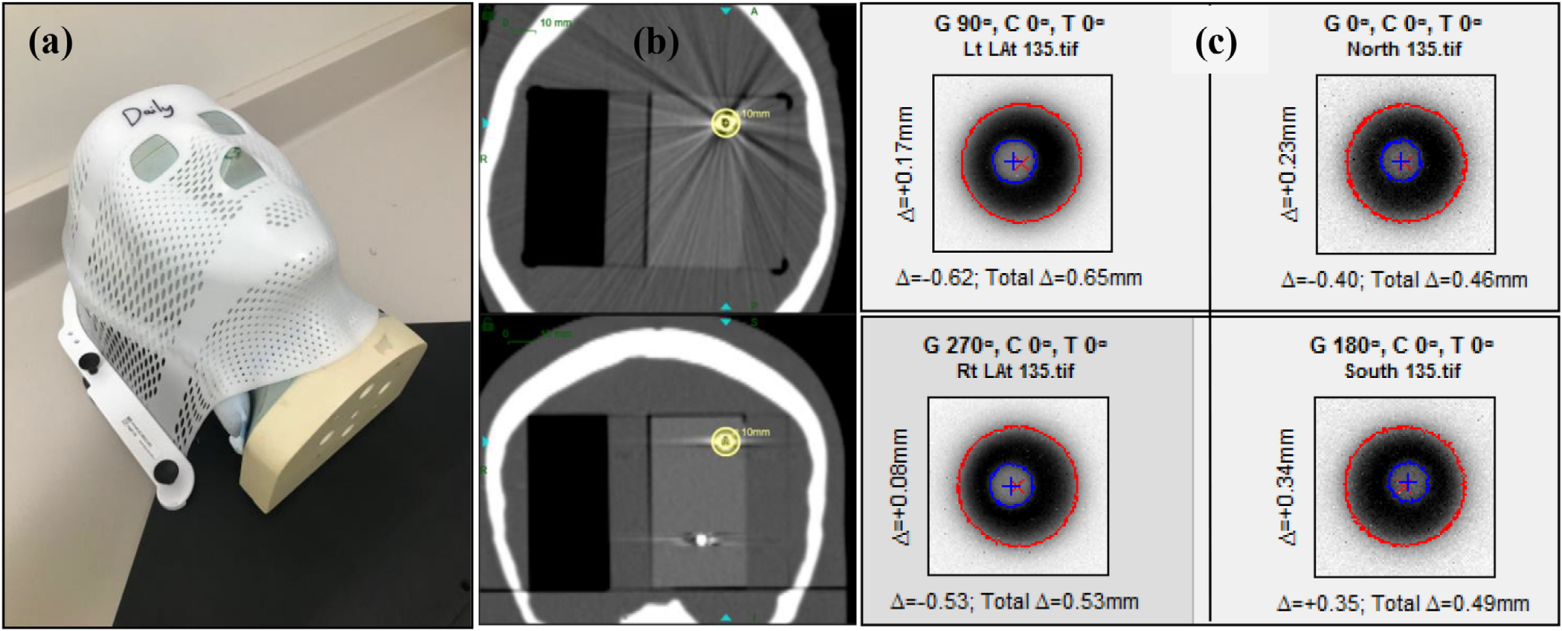
(a). The anthropomorphic phantom used for the Winston‐Lutz test. (b). CT image (axial and coronal planes shown) of the tungsten BB within the acrylic slab inserted in the head phantom in (a), with the 10 mm collimator (shown in yellow) centred on the BB. (c). Analysis of the extracted MV images at each cardinal gantry angle using DoseLab (Varian, Palo Alto, CA), to determine the congruence between the projection of the BB and the radiation field

The coincidence of the mechanical and radiation isocentre was tested using the WL test. A clear acrylic slab (80.90 mm × 80.65 mm, Standard Imaging, Inc., Middleton, WI, USA), embedded with four (4 mm diameter) tungsten BBs in a non‐coplanar arrangement was inserted in an anthropomorphic phantom (Figure 4a). The phantom was then CT scanned with 1 mm slice thickness and treatment plans were generated in the ZAP TPS to irradiate each BB isocentrically with the 10 mm collimator (Figure 4b) using the four cardinal gantry angles (0^0^, 90^0^, 180^0^, 270^0^). The phantom was positioned in the ZAP‐X system using the kV imaging system by matching the acquired X‐ray images with the DRRs. 200 MUs were delivered at each of the four cardinal gantry angles on the custom MV imager consisting of a scintillator with a Point Grey camera (Point Grey Research, Inc., Richmond, British Columbia, Canada). The images were extracted and uploaded into DoseLab (Varian, Palo Alto, CA, USA) to determine the congruence between the projection of the BB and the radiation field in the three translational directions (Figure 4c).

#### 2.2.2 Monthly end‐to‐end (E2E) tests

E2E tests for the ZAP‐X were conducted using the CK head phantom (Accuray, Sunnyvale, CA, USA) with Gafchromic EBT3 film (with slits) placed in the axial and sagittal planes within the Ball Cube II insert (Figure 3a and b). The phantom was CT scanned with 1 mm slice thickness using immobilization similar to that for a patient (i.e., head mask, accuform head rest). An isocentric treatment plan was generated in the ZAP TPS using the 25 mm collimator for a given pre‐set beam path (consisting of 36 beams) which generated a circular dose distribution centred on the hidden ‘ball’/target within the Ball Cube II insert. Minor positional adjustments were made to the isocentre placement to centre the 70% isodose line (IDL) on the ‘ball’ (as required by the Accuray's E2E software used for analysis) and 420 cGy was prescribed to the 70% IDL. The phantom was positioned in the ZAP‐X using kV imaging to match the CT simulation position, and the designed treatment plan was delivered. The irradiated films were scanned using an Epson Expression 10000 XL scanner (48‐bit colour, 300 dpi). The scanned films were analysed using Accuray's E2E software to obtain dose targeting errors in the translational directions, similar to what is done for E2E testing for a CK system.^26^ Results for the first ten E2Es were independently verified using ImageJ.

After the first 6 months, the procedure for monthly E2E tests was slightly modified. With the new process, an isocentric plan was generated for the same phantom configuration with a pre‐set beam path using the 10 mm collimator. The films were scanned with 48‐bit colour, 75 dpi parameters and film profiles (in the anteroposterior (A/P), lateral (R/L) and superior‐inferior (S/I) directions) were compared with the corresponding dose profiles extracted from the TPS in SNC patient software (Sun Nuclear Inc., Melbourne, FL, USA). Differences in the measured and calculated full width at half maximum (FWHM) were calculated in A/P and R/L using the axial film, and in A/P and S/I using the sagittal film. The square root of the sum of squared offsets in the three directions was used to calculate the total targeting error.

#### 2.2.3 Delivery quality assurance (DQA)

**FIGURE 5:**
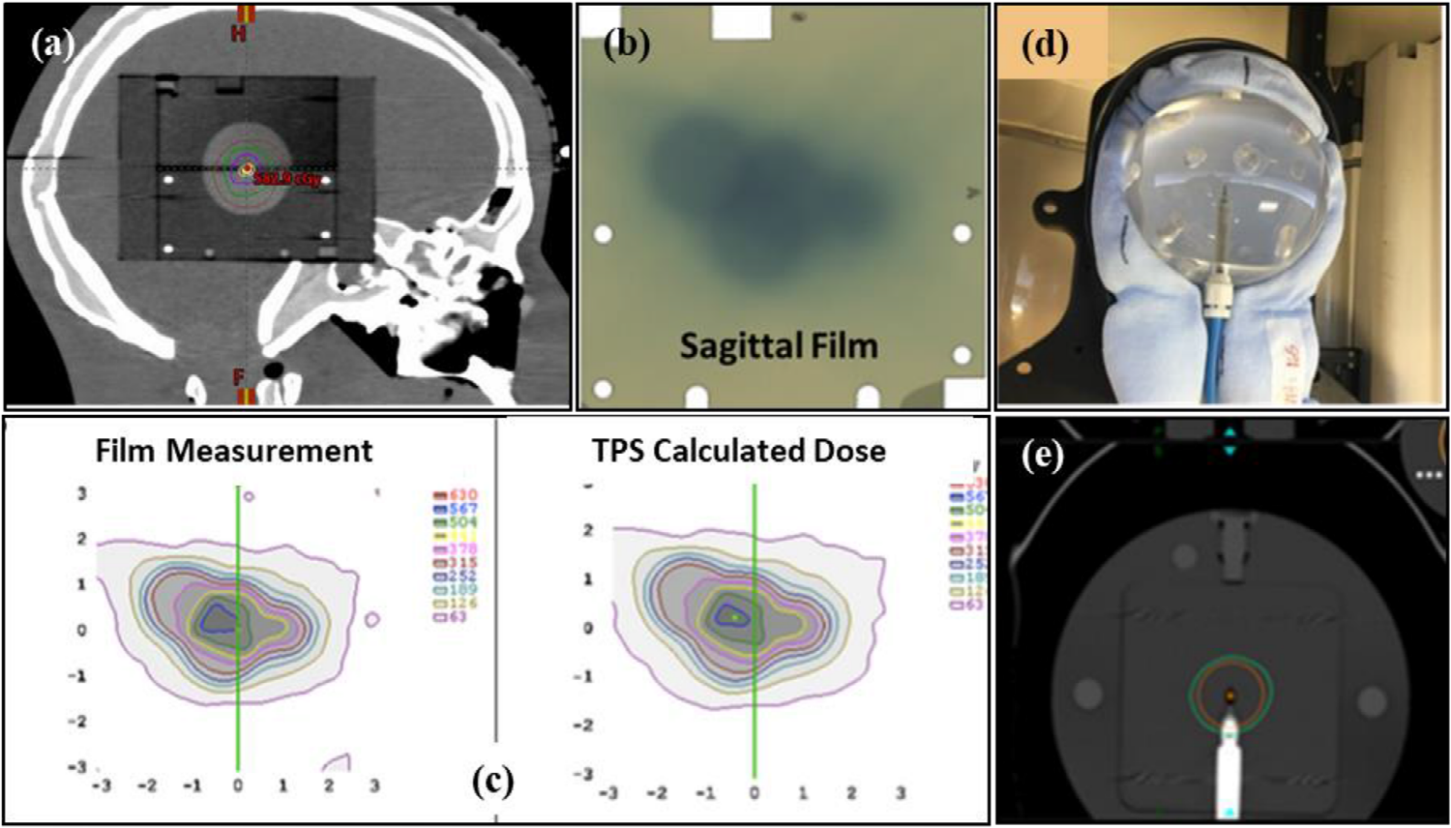
(a). A patient treatment plan overlayed on the head phantom CT scan with the dose distribution centred on the hidden target within the Ball Cube II insert. (b). An example of an irradiated DQA film. (c). Measured versus TPS calculated planar dose distributions for a DQA plan. (d). The setup for ion chamber based DQA measurements showing the Lucy phantom placed within an accufoam mold and with a PTW PinPoint chamber inserted. (e). An example DQA plan generated on a CT scan of the Lucy phantom shown in (d)

DQA was performed for each patient treated on the ZAP‐X unit using Gafchrmoic EBT3/XD film prior to treatment. The patient treatment plan was overlayed on a CT scan (1 mm slice thickness) of the head phantom by co‐registering the centre of the planning target volume in the patient's plan to the centre of the hidden target within the Ball Cube II insert (Figure 5a) using the ‘Plan QA’ feature available in the ZAP‐X TPS. 300 cGy was prescribed to the 50% isodose line for the DQA plans to keep the maximum dose at 600 cGy for those cases using EBT3 Gafchrmoic film. The MUs per beam were automatically scaled to meet this prescription dose. For DQA using XD films no MU scaling or modification to the clinical prescription was required.

Prior to delivering the QA plan, a no‐slit Gafchromic EBT/XD film was placed within the Ball Cube II insert in either the axial or sagittal plane. The head phantom was set up on the ZAP‐X treatment unit using a pre‐treatment imaging workflow, which compares planar kV images acquired at several gantry positions to DRRs generated from the TPS. Twenty four hours after plan delivery, the exposed films (Figure 5b) were scanned (48 bit colour, 75 dpi, no colour correction) using an Epson 10000XL flatbed scanner, after proper scanner warm up procedures while ensuring that the film scan direction was the same as that used for scanning the calibration films. Optical density values were converted to dose using a film calibration curve. Nine films were irradiated to specific doses ranging from 0 to 700 cGy (for EBT3) and 0 to 3000 cGy (for XD) on a TrueBeam (Varian, Palo Alto, CA, USA) linear accelerator (LINAC) for generating the calibration curve. Calibration films were irradiated at d_max_ in a solid water phantom, with a 6MV photon beam at 100 SSD for a 10 cm × 10 cm field size. The LINAC output on the day of film irradiation was used to determine the monitor units to be used for each film irradiation. SNC patient software was used to compare the measured film planar dose with the TPS calculated dose of the same slice/plane using gamma analysis (Figure 5c).

For the first five patients treated on the ZAP‐X, in addition to film‐based DQA, absolute point dose measurements were acquired with a PTW 31014 PinPoint (0.0015 cc) ion chamber (IC). The PinPoint IC was inserted into a 3D SRS Lucy phantom (Standard Imaging, Inc., Middleton, WI) which was immobilized using an accufoam mold (Figure 5d), and CT scanned with 1 mm slice thickness. A QA plan was generated for each patient treatment plan by centring the planning target volume on the IC active volume (Figure 5e). Prescription dose was kept the same as that in the actual treatment plan. The Lucy phantom with the IC inserted was positioned in the Zap‐X unit using kV imaging and the total charge measurement from the delivery of the QA plan was recorded using a PTW Unidos electrometer. The charge reading was converted to dose using the ADCL provided *N_D,w_* factor for the IC, while applying corrections for the temperature, pressure and measurement medium (a conversion factor of 0.97 was used given that the phantom is made of acrylic instead of water). Measured ion chamber doses were compared to the TPS‐calculated mean dose to the IC active volume.

### 2.3 Kilovoltage (kV) imaging system performance

The ZAP‐X integrated kV imaging system is attached to the linear accelerator and used for acquiring planar radiographic images for patient alignment and for monitoring intrafraction patient motion. The kV imaging system consists of a kV X‐ray tube and a flat panel detector. Image guidance is performed by matching acquired X‐ray images to DRRs. An initial set of near orthogonal alignment images are acquired for patient positioning. Additional images, at a user‐selected time interval (typically every 60 s) are then captured during treatment to verify alignment and to correct for any patient motion.

**FIGURE 6:**
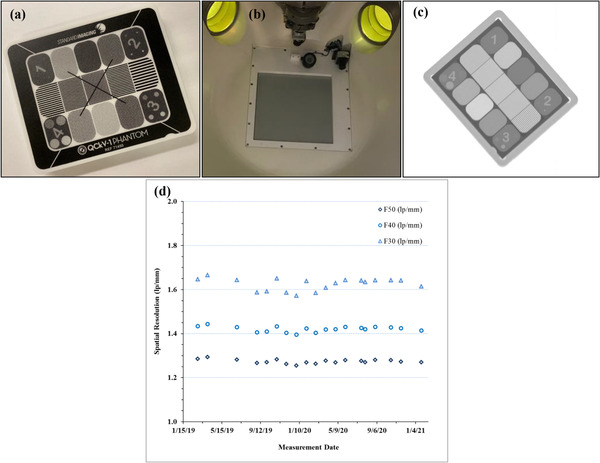
(a). The Standard Imaging QCkV1 phantom used for evaluating the quality of ZAP kV images. (b). The kV imaging panel on the ZAP‐X. (c). A kV image of the QCkV1 phantom acquired using the ZAP kV imaging panel (d). The change in spatial resolution of the kV imaging system based on MTF f_50_/f_40_/f_30_ over time

Performance of the kV imaging system and deviations from baseline (measurements acquired during commissioning) were evaluated using the QCkV1 phantom (Standard Imaging, Inc., Middleton, WI, Figure 6a). The phantom was directly placed on the centre of the kV imaging panel (Figure 6b) and an image was acquired (Figure 6c) using imaging parameters optimized particularly for this phantom (65 kV, 40 mA, 65 ms). A background image of the panel (without the phantom and with the couch moved out of the image) was also acquired using the same imaging technique. The corresponding DICOM images were analysed using PIPSpro Software (Standard Imaging, Inc., Middleton, WI, USA) for spatial resolution (modulated transfer function (MTF f_50_/f_40_/f_30_)) compared to baseline, as recommended per TG‐142.^9^ A visual inspection of the images was also performed and compared to baseline images.

## 3 RESULTS

**FIGURE 7:**
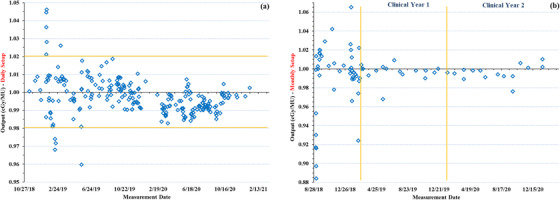
Beam output measurements acquired using (a) the daily setup and (b) the monthly setup, over a time period of more than 2 years. Horizontal yellow lines on (a), indicate the ± 2% institutional tolerance for daily output measurements. The vertical yellow lines in (b), separate the output data acquired into pre‐clinical, clinical year 1 and 2, to show the variation in output prior to the clinical use of the unit and thereafter

Beam output measurements acquired using the daily setup, spanning over 2 years (240 data points) are shown in Figure (7a). Prior to clinical use of the ZAP‐X, multiple output measurements (performed more than once a day) were carried out to establish optimal warmup procedures for daily output QA and to study the variation in machine output throughout the course of the day. The mean daily output across all measurements made was 0.997 ± 0.010 cGy/MU (range: 0.960–1.046 cGy/MU). Daily output on the days of clinical treatment were kept between ± 2% and any measurements that deviated by more than ± 2% were adjusted using the monthly output calibration protocol.

Variation in beam output (76 data points) measurements using the monthly setup since machine installation in late 2018 is presented in Figure (7b). Initially (prior to clinical use) output measurements with the monthly setup were performed more than once a month to evaluate machine performance, monitor variation in output with the number of monitor units delivered, and establish proper machine warmup procedures. Large variations in output (as high as 11.6%) were seen during this time period. Prior to clinical treatments, net cumulative output adjustments of ∼15% were made. Since the first patient treatment in late January 2019, overall output adjustments were ∼9% during the 1st year (2019) and ∼3% during the 2nd year (2020). The mean output was 0.993 ± 0.029 cGy/MU (range: 0.884–1.065 cGy/MU). Once the ZAP‐X was being used clinically, monthly output was adjusted when it deviated by more than ± 1% of 1 cGy/MU. As mentioned earlier, these monthly output measurements were performed with the same setup as that used for absolute dose calibration using the modified TG‐21 approach.

**FIGURE 8:**
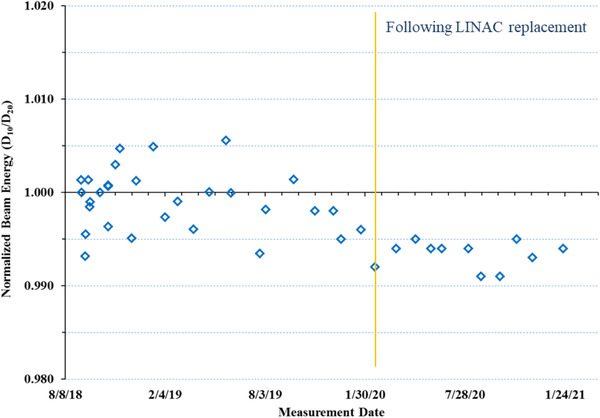
Relative change in the beam energy compared to baseline measurements over 2 years. The vertical yellow line indicates measurements made following linac replacement in early 2020

The annual absolute dose measurements carried out using a modified TG‐51 approach in water resulted in an output of 1 cGy/MU at d_max_ for a SAD of 450 mm. The third‐party independent verification of machine output using the SRS head phantom was within 3% with gamma pass rates of 98% (coronal) and 99% (sagittal) for 5%/3 mm gamma criteria for the 2D planar film dose measurements. The independent output verification using TLDs inserted in the GK ABS phantom was within 2% of our institutional measurement. The relative change in the beam energy compared to baseline measurements over 2 years (40 data points) ranged from 0.991 to 1.006 with a mean of 0.998 ± 0.004 (Figure 8). The flatness and symmetry of beam profiles based on film measurements along the ‘wheelplane’ and ‘orthoplane,’ were visually verified to align (Figure 3e) with TPS extracted beam profiles.

**FIGURE 9:**
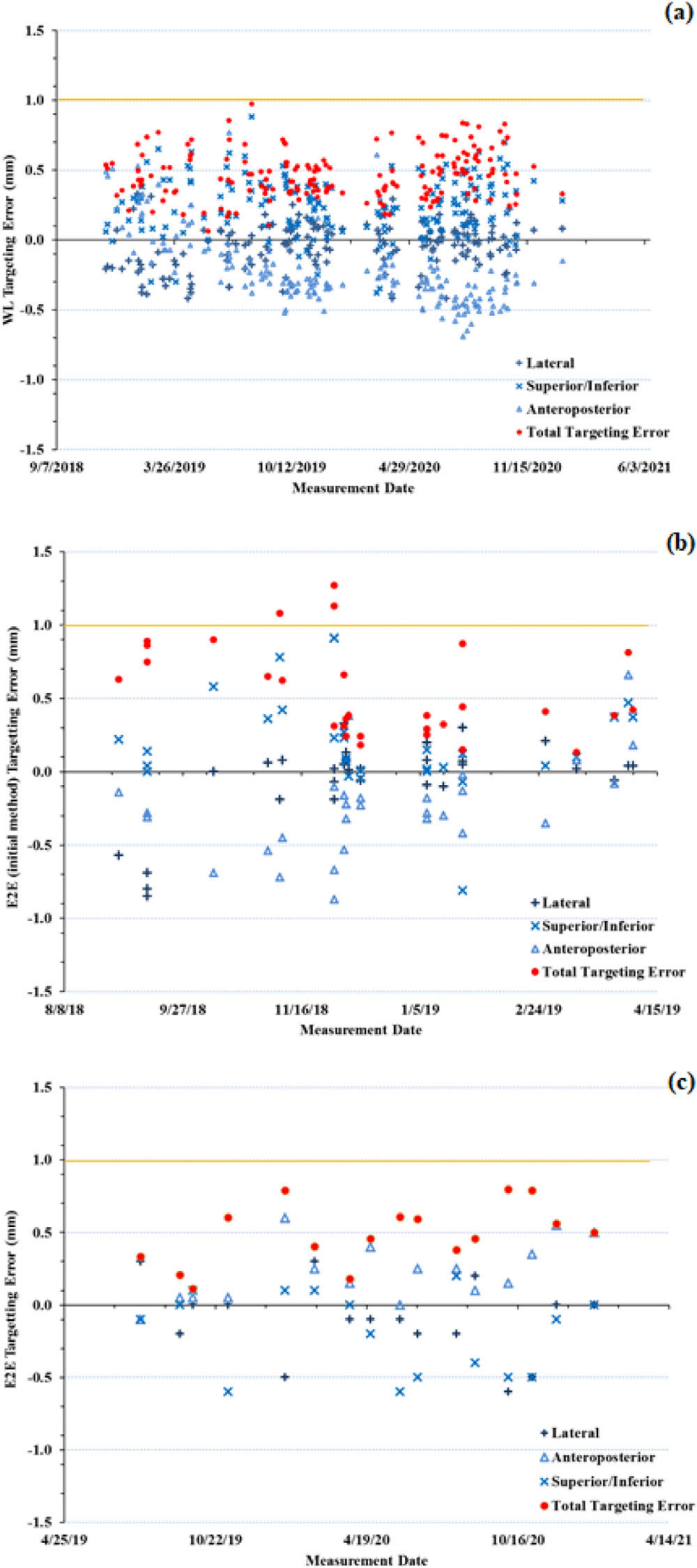
(a) Beam targeting error as quantified with the Winston‐Lutz test. The targeting error as quantified by the E2E tests as determined by (b) the initial 36 beam path E2E test performed in conjunction with Accuray's E2E software and (c) the E2E tests performed using a preset beam path with the 10 mm collimator, analysed using the SNC patient software. The total targeting error as determined by each method is shown in red, with the yellow horizontal line indicating the 1 mm tolerance for beam targeting. The data also shows the targeting error separated by translational direction

The daily WL test performed over the course of over 2‐years resulted in a mean deviation from isocentre of −0.04 ± 0.16, 0.24 ± 0.21 and −0.22 ± 0.26 mm toward patient right, superior and posterior directions (Figure 9a), respectively. The average total targeting error quantified with the WL test was 0.46 ± 0.17 mm (range: 0.06–0.98 mm). Initial E2E tests using the 36 beam path in conjunction with Accuray's E2E software (performed over approximately 6 months, 29 E2E tests) resulted in mean errors of −0.06 ± 0.29, 0.20 ± 0.33, and −0.21 ± 0.31 mm in the right, superior, and posterior directions, respectively (Figure 9b). Overall, the average total targeting error from these E2E tests was 0.54 ± 0.31 mm (range: 0.13–1.27 mm). Results for E2E tests analyzed using SNC patient over 1.5 years (Figure 9c) shows mean errors of −0.11 ± 0.26, 0.23 ± 0.21 and −0.19 ± 0.28 mm to the right, anterior and inferior directions, respectively. Overall, the average total targeting error from these monthly E2E tests was 0.49 ± 0.21 mm (range: 0.11–0.80 mm).

The average gamma passing rate for film‐based DQA was 99.02% (range: 94.00%–100%) for 2%/2 mm and 90.46% (range: 55.10%–100%) for 2%/1 mm gamma criteria for the first 80 patients treated on our ZAP‐X unit. IC‐based DQA performed for the first five patients, showed an agreement of 1.4% (range: 0.97%–2.84%) between the TPS calculated and IC measured mean dose within the chamber active volume. The change in spatial resolution of the kV imaging system over 2 years is shown in Figure (6d). The mean spatial resolution was 1.274 ± 0.009 (range: 1.255–1.294), 1.421 ± 0.012 (range: 1.396–1.444) and 1.625 ± 0.028 (range: 1.572–1.666) line‐pairs (lp)/mm for the MTF f_50_/f_40_/f_30_, respectively. Baseline values for MTF f_50_/f_40_/f_30_ were 1.285, 1.434 and 1.647.

## 4 DISCUSSION

Following completion of acceptance testing and commissioning^10^, ^12^, ^13^ of the ZAP‐X unit, quality assurance procedures were established for measuring beam output, energy, profile, targeting accuracy (WL and E2E), overall dose delivery (DQA) and performance of the imaging system. Baseline data was obtained and trends in the aforementioned parameters were kept track of over time to verify that the performance of the first clinical ZAP‐X unit were within standards and tolerances recommended by TG‐142^9^, TG‐135^8^ and MPPG 9a^27^ for SRS treatments.

Once the annual absolute dose calibration was performed, our institutional policy was to keep the daily and monthly beam output to within ± 2% and ± 1% respectively, prior to any clinical treatments. As such, anytime a deviation from these tolerances was seen the output was calibrated using the monthly output measurement process (TG‐21 approach) discussed earlier. Current, TG recommendations for beam output for a SRS unit is 3% (daily) and 2% (monthly) per TG‐142^9^ and 2% (both daily and monthly) per TG‐135^8^ and MPPG 9a.^27^ Given that the ZAP‐X was a novel treatment device, deviations in output above tolerance were seen on several occasions (Figure 7) throughout the course of the first year. In the early stages of output monitoring (prior to clinical use of the unit) we noticed the output drifting over 10%. To better stabilize the beam output, a new monitor chamber was installed in late 2018. Further, adjustments to the collimator wheel were made in mid 2019 and the original LINAC was replaced in early 2020 (Figure 7b, Clinical Year 2). Beam data was completely re‐acquired (water tank beam scanning^10^) or a subset of beam data was acquired and verified against TPS data as previously described by Srivastava et al.,^13^ following any changes that may have an impact on beam profiles/output. The cumulative net output adjustment prior to clinical treatments in 2019 was ∼15% while overall output adjustments during the 1^st^ and 2^nd^ clinical years of use were ∼ 9% and ∼3%, respectively. Change in output trending upward over time (due to changes within the monitor chambers), after the first few months following commissioning is common and has been reported on previously.^28^, ^29^, ^30^, ^31^ The stability of the ZAP‐X output from the time that the unit was in clinical use, is similar to what has been reported for other SRS treatment units (∼2%–5%).^30^, ^31^, ^32^, ^33^, ^34^, ^35^, ^36^ Change in our ZAP‐X beam energy over 2 years was within ± 1.0% and the change in beam profiles (acquired in a water tank for annual QA) was within 1% compared to the post processed beam data entered into the TPS, within the ± 2% tolerance for both recommended in TG‐135^8^ for robotic radiosurgery. The beam energy was lower by 0.5% percentage compared to the original baseline values following linac replacement in early 2020, and therefore not re‐baselined.

In terms of beam targeting, the total targeting error for daily WL tests were within 1 mm (mean: 0.46 ± 0.17 mm, range: 0.06–0.98 mm) and monthly E2E tests were all within 1 mm except for 3 data points (mean: 0.52 ± 0.28 mm, range: 0.11–1.27 mm) which were measured during the time the treatment unit was not in clinical use. Initially E2E tests were performed more than once a month to compare how beam targeting varied between the WL and E2E setups. If the total targeting error was 1 mm or more for the WL test or > 0.95 mm for the E2E test, multiple E2E tests were performed to calculate the isocentre offset in the three translational directions following TG‐135 recommendations for robotic radiosurgery. Corrections to the isocentre (or an isocentre shift similar to the delta‐man adjustment for CK) were applied to the planning CT DRRs within the ZAP treatment delivery software. Once initial beam targeting was established during acceptance and commissioning as described by Pinnaduwage et al.,^37^ adjustments to the isocentre needed to be performed twice during the course of 2‐years. Average total targeting errors observed for the ZAP‐X are similar to those reported for E2E tests performed on other SRS systems. Targeting errors of 0.4–0.5 mm have been reported for E2E tests on the CK system using the 6D skull tracking algorithm^26^, ^32^, ^38^ while mean 2D E2E targeting errors of 0.429 mm (maximum: 0.718 mm) and 0.225 mm (maximum: 0.284 mm) have been reported for framed and frameless GK.^39^

Film‐based DQA measurements for all ZAP‐X treatments were within the TG‐135 recommendation of a gamma pass rate of 90% for 2%/2 mm criteria. Average gamma pass rate results (averaging over all 80 DQA measurements) were 99.0% and 90.5% for 2%/2 mm and 2%/1 mm gamma criteria, respectively. A recent study^39^ characterizing a GK icon system, reported average gamma pass rates of 98.4% and 98.8% for framed and frameless/fractionated workflows for non‐clinical plans generated for two targets to verify E2E absolute dose distributions. DQA assessments on a VSI CK system^40^ performed for 150 patients, showed passing rates of 94.26% and 93.86% for 3%/1 mm and 2%/2 mm gamma criteria for target volumes ranging from 0.73 to 273.2 cc. Many studies have reported on the uncertainties of film‐based dose measuements^41^, ^42^, ^43^ with literature available for minimizing these uncertainties when performing film‐based DQA measurements for SRS/SBRT.^44^ For the DQA measurements discussed here, recommended procedures^41^, ^42^, ^43^ for film handling, irradiation and scanning were followed and the DQA results observed by our group for the ZAP‐X are comparable to those seen for other SRS systems.

Target volumes for cases treated on the ZAP‐X during the initial 2 years ranged from 0.03 cc to 26.51 cc (mean: 2.94 cc). Treatment prescriptions ranged from 14 to 20 Gy prescribed to the 50%–80% isodose line for single fraction treatments, and 25–30 Gy prescribed to the 50%–55% isodose line for five fraction treatments. The diagnoses for patients treated on the ZAP‐X were primarily brain metastases (46.6%) and meningioma (45.4%) with some cases of vestibular/acoustic schwannomas (6.8%), primary brain lesions (7.9%) and pituitary adenomas (1.1%).

## 5 CONCLUSION

This work discusses the stability and performance of the first clinical ZAP‐X unit, based on our initial 2‐year experience of using the unit focusing on beam output, energy and profile constancy, and beam targeting and dose delivery accuracy.

As is commonly seen with any new LINAC/monitor chamber, beam output was seen to fluctuate since commissioning, with output drifting by 9% and 3%, during the 1st and 2nd years of clinical use, respectively. For clinical treatments daily and monthly beam output was kept within ± 2% and ± 1%, respectively, per institutional policy. Change in beam energy was within ± 1% and the change in beam profiles was within 1% compared to the post processed beam data entered into the TPS. Over the initial 2 years, the average total targeting error based on daily WL tests was 0.46 mm and 0.52 mm based on the monthly E2E tests. Average gamma pass rate results for the 80 Gafchromic film‐based DQA measurements performed were 99.0% and 90.5% for 2%/2 mm and 2%/1 mm gamma criteria, respectively.

In terms of beam constancy, targeting and delivery QA, the ZAP‐X unit met tolerance limits recommended by TG‐135^8,^ MPPG 9a.,^27^ and TG‐142^9^ for SRS treatments. Further, in comparing beam constancy and targeting of the ZAP‐X with data available for other SRS systems in literature as well as our own experiences working with other dedicated SRS systems such as GK and CK, we conclude that the ZAP‐X is capable of delivering SRS treatments to a similar level of accuracy as what has been achieved with other SRS systems.

## AUTHOR CONTRIBUTION

All authors have collectively contributed in data collection, analysis, preparation and editing of this manuscript.

## CONFLICT OF INTEREST

No conflicts of interest.
